# Phosphaturic Tumor-Induced Osteomalacia

**DOI:** 10.7759/cureus.54712

**Published:** 2024-02-22

**Authors:** Diana Ashouri, Tony Kastoon

**Affiliations:** 1 Internal Medicine, University of California, Riverside, Riverside, USA; 2 Endocrinology and Obesity Medicine, University of California, Riverside, Riverside, USA

**Keywords:** fgf-23, hyperphosphaturia, hypophosphatemia, osteomalacia, tumor-induced osteomalacia

## Abstract

Tumor-induced osteomalacia (TIO) is a rare complication of certain tumors involving the skeletal bones, mainly in the lower extremities and rarely the spine, that can cause skeletal abnormalities, osteopenia, and osteoporosis. The etiology of these tumors is unknown, and they are considered benign tumors that usually localize in bone or soft tissue anywhere in the body. Symptoms are nonspecific and vague, which causes a delay in diagnosis. These tumors produce fibroblast growth factor-23, which causes hypophosphatemia due to renal wasting of phosphate and inhibits vitamin D3 activation, resulting in osteomalacia. The majority of these tumors are osteoblastic and rarely osteolytic. A PET scan can detect the location and diagnose these tumors. Surgical resection, when feasible, is the treatment of choice and can lead to improvement, resolution of symptoms, and correction of hypophosphatemia. Patients usually present with a wide variety of nonspecific complaints. This case report presents an unusual presentation of TIO from a phosphaturic mesenchymal tumor involving the left acetabulum.

## Introduction

Tumor-induced osteomalacia (TIO) is a rare condition associated with specific tumors, primarily affecting skeletal bones, often in the lower extremities. These tumors lead to skeletal abnormalities, osteopenia, and osteoporosis [1,2]. The exact cause is unknown, but these tumors are typically benign and can occur in various body tissues. TIO's vague and nonspecific symptoms often lead to delayed diagnosis. These tumors produce fibroblast growth factor-23 (FGF-23), causing hypophosphatemia and osteomalacia [1].

In this case report, we present an unusual case of TIO originating from a phosphaturic mesenchymal tumor in the left acetabulum, highlighting the diagnostic and management challenges of this rare condition.

## Case presentation

A 61-year-old Hispanic male with a past medical history of hypertension presented to the primary care physician with persistent generalized bone pain for two years. Initially, the pain was diffuse, mild in severity, and aggravated by motion. The patient denied any recent trauma or the intake of any new medications. The pain has gotten worse lately, especially in the lower extremities. He denied weight loss or fever. The physical examination was remarkable for multiple tender bone and joint points, but more so in the left hip area. Laboratory results showed normal blood count and chemistry, including creatinine, calcium, and vitamin D levels, but a low phosphorus level of 1.6 mg/dL (normal range: 2.5-5 mg/dL), and the vitamin D level was 31 ng/mL (normal range: 30-74 ng/mL). The urine phosphate level was 271 mg/dL (normal level: 25-125 mg/dL). A bone mineral density at both lumbar and femoral sites showed a loss in bone mass in the range of osteopenia (T-score lumbar spine: -2.1 g/cm², T-score at the femur neck: -1.5 g/cm²). Based on these findings, the patient was diagnosed with osteopenia and was treated symptomatically with vitamin D (5000 units daily), calcium (1000 mg), sodium phosphate tablets (1.5 g), and calcitriol (0.5 mcg).

**Figure 1:**
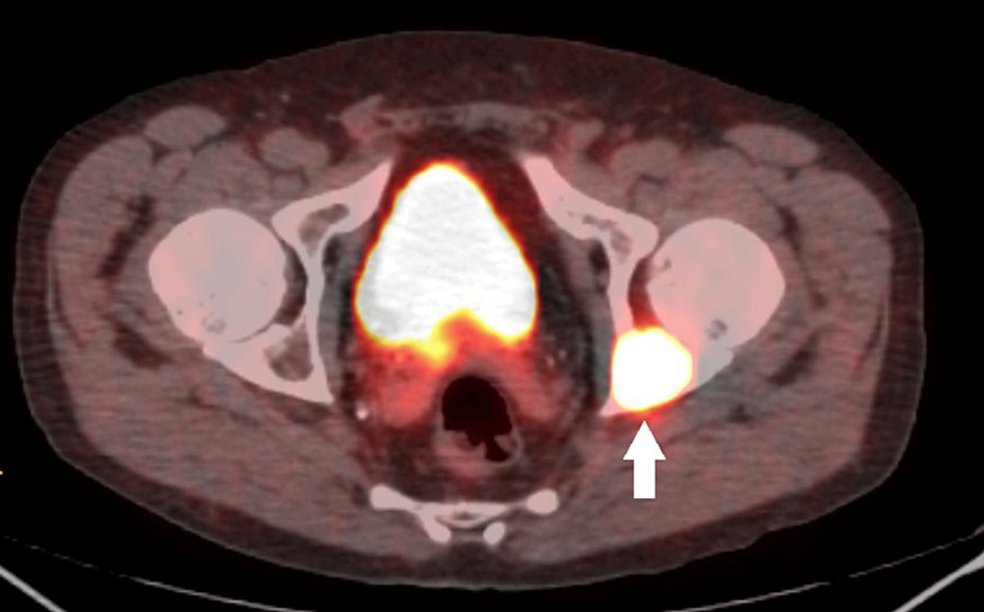
PET scan revealing hypermetabolic area in the left hip PET: positron emission tomography

**Figure 2:**
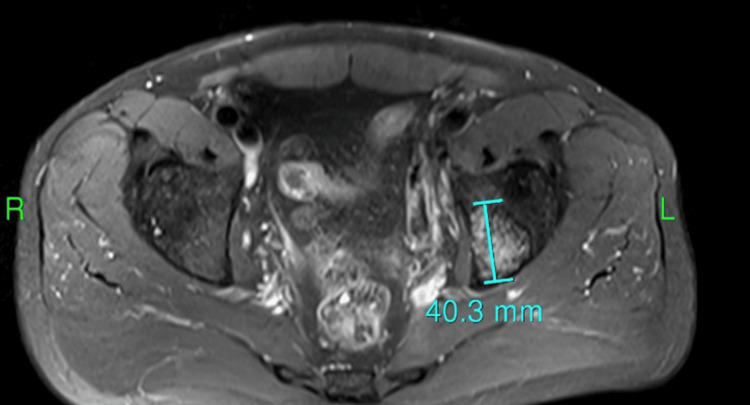
MRI illustration of bone tumor in the left hip acetabulum MRI: magnetic resonance imaging

**Figure 3:**
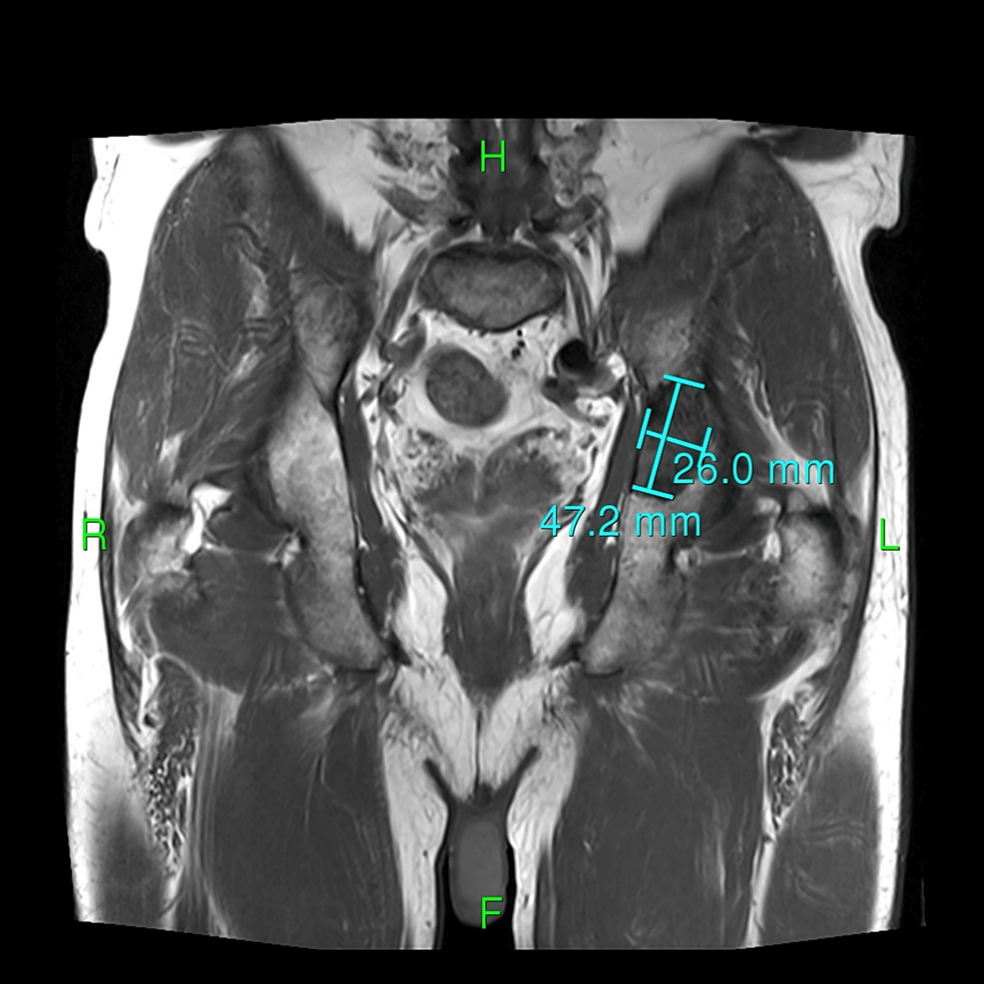
MRI coronal view of the left hip acetabulum tumor MRI: magnetic resonance imaging

After four weeks, the patient returned to the clinic with worsening bone pain, especially in the left hip. His phosphorus levels continued to decline to 1.7 mg/dL despite phosphorus supplements. The patient was referred to an endocrinologist, and further testing revealed an elevated FGF-23 level of 1650 RU/L (normal range: 54-180 RU/L). Genetic testing for X-linked hypophosphatemia revealed that no pathogenic variants associated with X-linked hypophosphatemia were identified in the genes analyzed, indicating a negative result. A positron emission tomography (PET) scan was conducted, which showed a hypermetabolic area in the left hip measuring 7.5 x 6.0 x 2.2 cm (Figure 1). There were no other hypermetabolic areas suggestive of metastasis. Subsequent magnetic resonance imaging (MRI) confirmed the presence of a bone tumor measuring 7.5 x 6.0 x 2.2 cm, involving the left hip acetabulum (Figures 2-3). This tumor was surgically excised, and pathology showed a mesenchymal spindle cell tumor without any vascular invasion. Three weeks after resection, bone pain subsided, and the phosphorus level became normal (2.5 mg/dL). The patient continues to do well one-year post-resection.

## Discussion

Mesenchymal stromal cells (MSCs) are mainly present in bone marrow and other tissues. They represent a small and heterogeneous subpopulation of mesenchymal stem cells that possess multilineage differentiation potential [3]. These cells have the ability to differentiate into cells of mesodermal origin, such as adipocytes, chondrocytes, osteoblasts, or fibroblasts [4]. Tumors that have predominant MSCs are unique in their features and presentations [2]. Calcium and phosphorus abnormalities associated with osteomalacia are the predominant presentation of these tumors due to their direct effect on bone [3]. Tumors are considered benign in nature [5].

Herein, we present a rare case of mesenchymal TIO of the left acetabulum presented with debilitating bone pain and muscle weakness. The patient initially presented with fatigue and vague diffuse bone pain that was nonspecific and associated with hypophosphatemia and did not respond to phosphate and calcium supplements. Bone density tests showed osteomalacia.

TIOs are paraneoplastic tumors that produce a variety of factors and hormones that cause nonspecific findings [2]. The differential diagnosis initially included X-linked hypophosphatemia and intrinsic kidney diseases [6]. TIOs are generally very rare tumors that present with symptoms similar to those of other chronic conditions, such as hypothyroidism, X-linked hypophosphatemia, and osteoporosis. It appears that the majority are considered to be phosphaturic mesenchymal tumors (65-75%), followed by hemangiopericytoma (6.3-9.1%), giant cell tumor (2.6-2.9%), and hemangioma (2.1%) [3]. The differential diagnosis initially included X-linked hypophosphatemia and intrinsic kidney diseases.

The etiology is unknown, and its incidence is estimated to be fewer than five persons (<0.008 cases per 100,000) [3]. Diagnosis is always delayed because of the nonspecific symptoms. Most cases are diagnosed after excluding other diseases [3]. The mean time to diagnosis is estimated to be 2.9 years [3]. The lack of serum phosphate measurement in many standard comprehensive chemistry panels contributes to its delayed diagnosis. In addition, the causative tumor can develop anywhere in the body and can be small enough to elude even our modern imaging techniques [7]. Hypophosphatemia due to elevated FGF-23 is the predominant laboratory finding [3].

The tumor produces an excessive amount of FGF-23, causing bone absorption, inactivation of vitamin D3 activation, and shutting down the kidney sodium-phosphate excretion process at the proximal tube, leading to a phosphate-wasting syndrome [3]. TIOs are predominantly osteoblastic, with 95% being osteoblastic and 5% osteoclastic [3]. PET and CT imaging are the most sensitive and specific tools for diagnosis [3]. They will detect the location, size, and metabolic features of the tumor. These tumors usually present as hypermetabolic lesions. TIOs affect predominantly the lower extremities (42%), craniofacial area (21%), hip and pelvis (12%), abdomen, thorax, and neck (11%), and very rarely affect the spine (12%) [7].

Once the diagnosis is established and the tumor is located, a complete resection of the tumor is the only definitive curative treatment [3]. Surgical resection leads to a cure in 72.7% of the cases. Tumor recurrence occurs in 9% of the cases. A biopsy is not needed unless the features of the tumor are suggestive of malignant bone cancer [2]. Upon complete resection, all laboratory abnormalities get corrected within one to two weeks [3], and FGF-23 levels become normal within days of surgery. Symptoms start improving within a few days of surgery and completely resolve within weeks of resection [5]. It is unknown how long it takes to improve the osteopenia. Patients need to be followed closely after surgery with electrolyte monitoring until it is normalized and then every six months [3]. For patients who are not surgical candidates or in cases of an undetected tumor, medical treatment using phosphate and calcitriol replacement is warranted [3]. Monoclonal antibodies against FGF-23, such as burosumab, have been widely used now in these patients with a good response rate in patients' symptoms and phosphate [2]. The overall prognosis is excellent, even with recurrence.

## Conclusions

TIOs have variable forms of presentation but are overall considered benign. Establishing an early diagnosis is critical and can often be accomplished by performing a PET scan for any patient presenting with unexplained osteomalacia associated with phosphaturia, elevated FGF-23, and bone pain. Complete resection is curative in surgical candidates, and when surgery is not feasible, medical treatment using burosumab is indicated.

## References

[1] Abramson M, Glezerman IG, Srinivasan M, Ross R, Flombaum C, Gutgarts V (2021). Hypophosphatemia and FGF23 tumor-induced osteomalacia in two cases of metastatic breast cancer. Clin Nephrol.

[2] Bosman A, Palermo A, Vanderhulst J (2022). Tumor-induced osteomalacia: a systematic clinical review of 895 cases. Calcif Tissue Int.

[3] Abrahamsen B, Smith CD, Minisola S (2021). Epidemiology of tumor-induced osteomalacia in Denmark. Calcif Tissue Int.

[4] Florenzano P, Hartley IR, Jimenez M, Roszko K, Gafni RI, Collins MT (2021). Tumor-induced osteomalacia. Calcif Tissue Int.

[5] Jiang Y, Xia WB, Xing XP (2012). Tumor-induced osteomalacia: an important cause of adult-onset hypophosphatemic osteomalacia in China: report of 39 cases and review of the literature. J Bone Miner Res.

[6] Minisola S, Peacock M, Fukumoto S, Cipriani C, Pepe J, Tella SH, Collins MT (2017). Tumour-induced osteomalacia. Nat Rev Dis Primers.

[7] Paquet M, Gauthé M, Zhang Yin J (2018). Diagnostic performance and impact on patient management of (68)Ga-DOTA-TOC PET/CT for detecting osteomalacia-associated tumours. Eur J Nucl Med Mol Imaging.

